# How *Streptomyces anulatus* Primes Grapevine Defenses to Cope with Gray Mold: A Study of the Early Responses of Cell Suspensions

**DOI:** 10.3389/fpls.2017.01043

**Published:** 2017-06-28

**Authors:** Parul Vatsa-Portugal, Aziz Aziz, Marine Rondeau, Sandra Villaume, Hamid Morjani, Christophe Clément, Essaid Ait Barka

**Affiliations:** ^1^Laboratoire de Stress, Défenses et Reproduction des Plantes, UFR Sciences Exactes et Naturelles, Unité de Recherche Vignes et Vins de Champagne EA 4707, Université de Reims Champagne-Ardenne, ReimsFrance; ^2^MEDyC – CNRS UMR7369, Faculty of Pharmacy, University of Reims Champagne-Ardenne, ReimsFrance

**Keywords:** *Actinobacteria*, *Botrytis cinerea*, defense responses, early signaling, grapevine

## Abstract

Gray mold, caused by *Botrytis cinerea*, is one of the most destructive diseases of grapevine and is controlled with an intense application of fungicides. As alternatives to chemicals, beneficial microbes may promote plant health by stimulating the plant’s immune system. An actinomycete, *Streptomyces anulatus* S37, has been screened from the rhizosphere microbiome of healthy *Vitis vinifera* on the basis of its ability to promote grapevine growth and to induce resistance against various phytopathogens, including *B. cinerea*. However, molecular mechanisms involved locally after direct perception of these bacteria by plant cells still remain unknown. This study focuses on local defense events induced in grapevine cells during interactions with *S. anulatus* S37 before and after pathogen challenge. We demonstrated that *S. anulatus* S37 induced early responses including oxidative burst, extracellular alkalinization, activation of protein kinases, induction of defense gene expression and phytoalexin accumulation, but not the programmed cell death. Interestingly, upon challenge with the *B. cinerea*, the *S. anulatus* S37 primed grapevine cells for enhanced defense reactions with a decline in cell death. In the presence of the EGTA, a calcium channel inhibitor, the induced oxidative burst, and the protein kinase activity were inhibited, but not the extracellular alkalinization, suggesting that Ca^2+^ may also contribute upstream to the induced defenses. Moreover, desensitization assays using extracellular pH showed that once increased by *S. anulatus* S37, cells became refractory to further stimulation by *B. cinerea*, suggesting that grapevine cells perceive distinctly beneficial and pathogenic microbes.

## Introduction

Plant innate immunity is a potential basal defense system, which provides powerful weapons to the host plants to fight against invaders. Beneficial bacteria or derived elicitors referred to as microbial-associated molecular patterns (MAMPs) have been identified as alarm/danger signals to trigger the plant innate immune responses (Jones and Dangl, 2006). In addition, they are able to activate the second line of defense mechanisms leading to induced systemic resistance (ISR) from a pathogen. The perception of some of these beneficial microbes involves early responses including ion fluxes, MAP kinase cascade activation, extracellular medium alkalinization, and the production of reactive oxygen species (ROS) (Van Loon et al., 2008; Verhagen et al., 2010; Bordiec et al., 2011). These events are followed by an activation of various molecular and cellular host-defense responses namely, defense-related gene expression, phenolic accumulation, lignin and callose deposition as well as hypersensitive response (HR) (Jones and Dangl, 2006; Bordiec et al., 2011; Verhagen et al., 2011; Farace et al., 2015). The induced defense responses are often linked to an activation of a complex signal transduction network, which involves mainly salicylic acid (SA), jasmonic acid (JA), and/or ethylene (ET) as important regulators of plant immunity (Pieterse et al., 2012), depending on pathosystems (Thomma et al., 1998; Kunkel and Brooks, 2002). The role of these signaling pathways in the regulation of ISR was pointed out in many plant-beneficial microorganisms interaction (Pieterse et al., 2014), supporting the idea that JA and ET are dominant players in the regulation of SA-independent systemic immunity conferred by beneficial microorganisms. In response to pathogen attacks, the production of ROS is also of considerable importance in plant defense (for review, Heller and Tudzynski, 2011). ROS are considered as second messengers, inducing several resistance responses including synthesis of pathogenesis-related proteins and phytoalexins, and programmed cell death in neighboring cells (Torres et al., 2006). ROS are also directly detrimental to pathogens, which prevent further disease spread (Torres and Dangl, 2005). In the host plant, the intensity of the induced oxidative burst correlates with the aggressiveness of *Botrytis cinerea* (Tiedemann, 1997). Further, instead of suppressing the plants oxidative burst, *B. cinerea* seems to exploit and might even contribute to this defense reaction (Govrin and Levine, 2000).

The production of H_2_O_2_ has also been assumed to be one of the most important signal molecules that could be linked to the development of ISR in whole plants (Van Loon et al., 2008; Verhagen et al., 2010; Bordiec et al., 2011; Farace et al., 2015). H_2_O_2_ can diffuse freely across membranes and, therefore, has been implicated in the signal transduction and the activation of defense responses (Aziz et al., 2004). This can lead to the cell wall protein cross-linking, thereby strengthening the cell wall (Lamb and Dixon, 1997).

The rapid activation of MAP kinase cascades is also involved in various signaling and regulatory mechanisms as well as alterations in the expression of several defense genes, which lead to the plant resistance. Most research in innate immunity gravitates toward MAMPs than live beneficial bacteria. In *Arabidopsis*, flagellin perception is transduced through a MAP kinase cascade (Asai et al., 2002) resulting in enhanced resistance against *Pseudomonas syringae* pv. tomato, which was associated with a callose deposition and an activation of *PR* genes (Gómez-Gómez et al., 1999). Similarly, pretreatment of tobacco leaves with lipopolysaccharides from the non-pathogenic *Burkholderia cepacia* was associated with the phosphorylation of an ERK-like MAP kinase and enhanced protection against *Phytophthora nicotianae* (Piater et al., 2004). Some non-pathogenic rhizobacteria-derived MAMPs primed plants for enhanced defense reactions upon challenge with a pathogen without extensive transcriptional reprogramming or cell death (Pieterse et al., 2014). In grapevine, Gruau et al. (2015) have reported that *Pseudomonas fluorescens* PTA-CT2-mediated ISR was accompanied by a down-regulation of *HSR* gene, a marker of HR/cell death after the *B. cinerea* challenge.

Another important response in the grapevine is the accumulation of stilbenic phytoalexins, especially *trans*-resveratrol (3,5,4′-tryhydroxystilbene) and its oligomer, *trans*-𝜀-viniferin during plant–microbe interactions (Delaunois et al., 2009; Jeandet et al., 2014). These stilbenic compounds are selectively accumulated in leaves and grape cell suspensions in response to various rhizobacteria (Verhagen et al., 2010; Aziz et al., 2016), and were shown to be associated with resistance of plants to pathogens.

Research aimed at understanding induced resistance mechanisms has scarcely elucidated in grapevine plants. For instance, pretreatment of grapevine plants with *Burkholderia phytofirmans* PsJN, *Pseudomonas* sp., *Pantoea* sp., or *Acinetobacter* sp. improve the grapevine resistance to subsequent infection with *B. cinerea* by eliciting defense-responses, including a stimulation of chitinases and β-1,3-glucanases activities, accumulation of phytoalexins, and an induction of defense genes (Compant et al., 2008; Trotel-Aziz et al., 2008; Gruau et al., 2015). *Streptomyces* is another important and versatile genera of *Actinobacteria* (Ait Barka et al., 2016) that may impact plants growth promotion by affecting their metabolism (Salla et al., 2014). In addition, *Streptomyces* sp. can also induce a local and a systemic resistance in grapevine and *Arabidopsis* to pathogens (Conn et al., 2008; Loqman et al., 2009; Couillerot et al., 2014). The *Streptomyces*-induced resistance in *Arabidopsis* seems to be dependent on SA but not on JA/ET pathway (Conn et al., 2008).

*Streptomyces anulatus* S37 isolated from the rhizosphere of healthy wild *Vitis vinifera* have been shown as an endophytic plant growth promoting bacteria that enhances disease resistance against several pathogens including *B. cinerea* (Loqman et al., 2009). However, despite their putative importance for biocontrol and/or growth stimulation, the cellular and molecular mechanisms involved in the perception of this bacterium by plant cells still remain unknown.

Many aspects of the defense response can be observed in suspension-cultured plants treated with substances of fungal or bacterial origin, so called-elicitors. Therefore, cultured plant cells have been widely used as model systems to study the recognition and the transduction of microbial signals as well as the defense response itself (Dixon and Lamb, 1990). While the systemic induction of resistance by beneficial bacteria is well understood, little data are available on local defense events taking place during the interaction between grape cells and these bacteria. Additionally, no direct comparison has been made between defense responses prompted by actinomycete bacteria and the typical defense reactions occurred during non-host or incompatible interactions triggered by *B. cinerea*.

Herein, our objective is to understand how plants could integrate signals induced by beneficial *S. anulatus* S37 into an immune response that maximizes both profitable and protective functions against the necrotrophic fungus *B. cinerea*. This will be accomplished by underlying the local defense events induced by *S. anulatus* S37 after their perception by grapevine cells, and after infection with *B. cinerea*. Our results indicate that *S. anulatus* S37 was perceived by grapevine cells by triggering early and late responses including oxidative burst, extracellular alkalinization, activation of protein kinases, induction of defense genes expression and phytoalexin accumulation, but not the programmed cell death. Further, upon challenge with *B. cinerea*, the *S. anulatus* S37 primed grapevine cells to enhanced defense reactions and reduced the pathogen-induced cell death. Once stimulated by the bacterium, plant cells became refractory to further stimulation by *B. cinerea*, suggesting a different mode of perception of beneficial and pathogenic microbes by grapevine cells.

## Materials and Methods

### Plant Cell Culture

Concord grape (*Vitis labrusca*) cell suspensions were cultured in Murashige and Skoog (MS) medium (pH 5.8) containing vitamins (×1.5), sucrose (30 g l^-1^), 2,4-dichlorophenoxyacetic acid (2,4-D, 0.2 mg l^-1^), 6-benzylaminopurine (BAP, 0.5 mg l^-1^) and were propagated in the dark at 25°C under shaking at 120 rpm. They were sub-cultured every 7 days to be maintained in exponential phase. For experiments, 30 ml of cells sub-cultured for 7 days were collected and resuspended in fresh MS medium for 24 h before treatment (Aziz et al., 2004).

### Microorganisms

Bacteria were collected by centrifugation (3,000 × *g* for 15 min) and washed twice with a phosphate-buffered saline (PBS) (10 mM, pH 6.5). The pellet was resuspended in the PBS and used as inoculum. The bacterium *S. anulatus* S37 concentration was estimated by the spectrophotometer (600 nm) and adjusted to 10^6^ colony forming units per ml (cfu ml^-1^) with the PBS (Loqman et al., 2009).

*Botrytis cinerea* strain 630 used in this study was provided by Dr. Brygoo (INRA, Versailles, Grignon, France) and maintained on the potato dextrose agar (PDA, Difco, United States). The *B. cinerea* inoculum was initiated by growing the fungus on fresh PDA medium to obtain abundant hyphal swellings. After 3 weeks, conidia collected from the PDA medium with sterile distilled water were filtered through a sterile 25 μm filter. The density of *B. cinerea* conidiospores was then determined and adjusted to 10^5^ spores ml^-1^ for all bioassays.

### Cell Treatments

Cells were collected during the exponential growth phase and washed by filtration in a suspension buffer containing 175 mM mannitol, 0.5 mM K2SO4, 0.5 mM CaCl2, and 2 mM MES, pH 5.5. Cells were resuspended at 0.1 g FW ml^-1^ with a suspension buffer and equilibrated for 2 h on a rotary shaker (120 rpm, 25°C). Grapevine cells were then used to analyze the extracellular pH, the H_2_O_2_ production, the MAP kinase assay, the cell death, the defense-related gene expression, and the phytoalexin production after a treatment with the *S. anulatus* S37 and/or the *B. cinerea.* Control cells were incubated under the same conditions without treatment. The EGTA (Sigma) was supplied at 3 mM, for 10 min, before an inoculation with the S37 and/or the *B. cinerea*.

### Determination of Extracellular pH and Hydrogen Peroxide

The extracellular pH variation was analyzed according to Felix et al. (1993) in 10 ml of agitated cell culture using a glass pH electrode (Basic, Denver Instrument, Gottingen, Germany). The production of H_2_O_2_ was analyzed by chemiluminescence from the ferricyanide-catalyzed oxidation of luminol using a luminometer (Lumat LB 9507, Berthold) as described previously (Aziz et al., 2004). The chemiluminescence was integrated and expressed as nmol H_2_O_2_ per g FW, using a standard calibration curve with H_2_O_2_.

The refractory state experiments on grapevine cell suspensions were analyzed by the extracellular pH change after a successive addition of the *S. anulatus* S37 and the *B. cinerea*. Cells were first treated at time 0 with the *S. anulatus* S37 at 10^6^ cfu ml^-1^ or with the *B. cinerea* at 10^5^ spores ml^-1^, washed at 50, 100, and 150 min with a fresh medium, and then treated, at 150 min, a second time with the *S. anulatus* S37 or the *B. cinerea* at the same concentrations.

### In-gel Protein Kinase Assay

*In vivo* experiments followed by in-gel kinase assays were performed as previously described (Vandelle et al., 2006) using myelin basic protein (MBP) as a MAP kinase substrate at the final concentration of 0.2 mg ml^-1^.

### Detection of Cell Death

The cell death was visualized by the FDA (fluorescein diacetate; Sigma–Aldrich) staining. Fresh cells were incubated in FDA (10 μg ml^-1^ of PBS) in the dark to maximize the formation of the fluorescein. The fluorescent FDA signals were detected with a fluorescence microscope (Olympus BX 51, Olympus, Japan). Only cells that exhibited bright green fluorescence from their cytosol were considered to be viable.

The cell death was also evaluated by analyzing caspase-like activity in grapevine cell suspensions, using the Muse MultiCaspase-7-AAD Assay as described by the manufacturer (Millipore, Molsheim, France). The assay determines simultaneously the percentage of cells showing a caspase activity and cell death fraction. Briefly, after each experimental condition, cells are resuspended at a density of 2–5 × 10^5^ cells ml^-1^ in the 1X Assay Buffer. The 50 μl of cell suspension were then added to each measurement tube. Then, 5 μl of Muse^TM^ MultiCaspase Reagent working solution were added to each tube. After vortexing at a medium speed for 3 to 5s, tubes were incubated for 30 min in the 37°C incubator with 5% CO_2_. Subsequently, 150 μl of Muse^TM^ 7-AAD working solution were added to each tube. After vortexing as indicated before, tubes were incubated at room temperature for 5 min, protected from light. After cell analysis, percentages of gated cells were calculated. Data indicate viable cells without caspase activity, cells exhibiting caspase activity without death marker, cells in the late stages of caspase activity with death marker, and cells that have died *via* necrosis but not through the caspase pathway.

### RNA Extraction and Quantitative RT-PCR Analysis

Cells were ground in the liquid nitrogen and total RNA was extracted from 50 mg of ground powder following the Concert PlantRNA reagent protocol according to the manufacturer’s instructions (Life Technologies). The RNA pellets were resuspended in 30 μl of RNAse-free water and incubated 2 h at -20°C for solubilization. The genomic DNA was removed by a DNAse treatment according to the manufacturer’s instruction (RQ1 RNase-Free DNase – Promega). The first-strand cDNA was synthesized from 150 ng of total RNA using the Verso cDNA Synthesis kit (Thermo Scientific). The quantitative RT-PCR was performed as described in Gruau et al. (2015), with Absolute Blue QPCR SYBR Green ROX Mix (Thermo Scientific) using a CFX96 system thermocycler (Bio-Rad). PCR reactions were carried out in duplicates in 96-well plates in a 15 μl final volume containing Absolute Blue SYBR Green ROX mix (including Taq polymerase, dNTPs, and SYBR Green dye), 280 nM forward and reverse primers and 30-fold diluted cDNA. The specificity of PCR reaction was also checked and *EF1α* and *60RSP* genes were used as internal controls for the normalization (Gruau et al., 2015). Results are expressed as the folds increase of transcript level relative to untreated cells as the control sample (1x expression level). The gene-specific primers (Supplementary Table S1) were designed based on sequences present in databases (Gruau et al., 2015).

### Phytoalexin Extraction and Analysis

Stilbenic phytoalexins were extracted from 200 mg of freeze-dried cell powder with 2 ml of methanol:water 85% v/v. Tubes were placed in the shaker for 1 h at the room temperature and then centrifuged for 10 min at 8000 ×*g*. The supernatant was dried under a nitrogen stream and residues were solubilized with 1 ml of methanol, filtered through 0.22 μm PTFE filters. Stilbenes were analyzed using an Acquity^TM^ UPLC system (Waters Corporation, Milford, CT, United States) as described in Hatmi et al. (2014), identified and quantified with reference to retention time and calibration with external standards.

### Statistical Analyses

Each experiment was repeated at least three times, unless indicated elsewhere. For gene expression, medium alkalinization, H_2_O_2_ production and phytoalexin production, results are expressed as the mean ± SE of a triplicate from one representative experiment out of three independent experiments (in each experiment three different extractions were pooled). For cell death, MAP kinase and Caspase-like activity, the data have been presented as a representative of three independent experiments. The refractory state experiment, reported data mean ± SE of duplicates, representative of two independent experiments. All collected data were submitted to ANOVA and the significance of differences among treatments was recorded at *p* < 0.05.

## Results

### Early Events Induced by *S. anulatus* S37 in Grapevine Cell Suspensions

#### Extracellular Alkalinization

**FIGURE 1:**
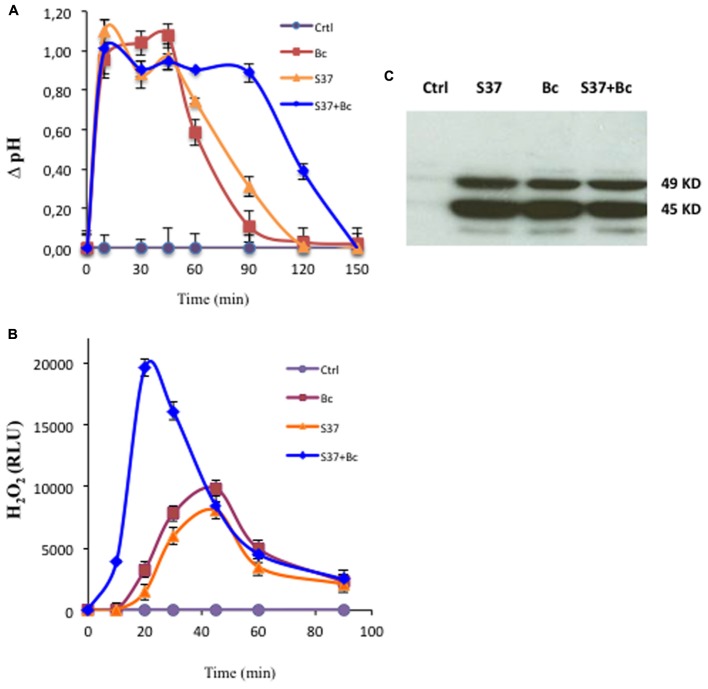
Early events in grapevine cell suspensions. Medium alkalinization **(A)**, accumulation of H_2_O_2_ **(B)** or MAP kinase activity **(C)** in control (circle), cells challenged with *B*. *cinerea* (square), with S37 (triangle), and with S37 + *B*. *cinerea* (diamond). MAPK activity was performed after 15 min of treatment. Data presented are means of triplicate experiments ± SD. For MAPK, data presented are representative of triplicate experiments. Bc, *Botrytis cinerea;* S37, *S*. *anulatus* S37.

The alkalinization of the extracellular medium is a useful tool for analyzing rapid events known to mediate MAMP-induced defense responses (Felix et al., 1998; Küpper et al., 2001). Grapevine cell suspensions treated with *S. anulatus* S37, *B. cinerea*, and S37 + *B. cinerea* showed an increase in the pH of the medium by 1.1 unit within 10 min. The pH variation was a transient and almost similar when cells were treated with *S. anulatus* S37 or *B. cinerea* (**Figure 1A**), returning to the basal value after 120 min. However, in the presence of S37 + *B. cinerea*, the medium alkalinization was maintained longer that individual treatment and came down to a basal level only after 150 min.

#### Production of H_2_O_2_

Oxidative burst is one of the early events that generally assumed to be involved in the regulation of induced plant immune responses (Aziz et al., 2004; Torres, 2010; Zipfel and Robatzek, 2010). Here, grapevine cell suspensions were inoculated with *S. anulatus* S37 and/or *B. cinerea*, and the released H_2_O_2_ was quantified. As shown in **Figure 1B**, grapevine cells responded to S37 and *B. cinerea* with transient and similar release of H_2_O_2_. For both, the oxidative burst was detected approximately after 10 min and peaked 45 min after the inoculation. Thereafter, the H_2_O_2_ concentration in the medium declined. When grapevine cells were inoculated with S37 + *B. cinerea*, a high burst of H_2_O_2_ was observed, which was almost seven-fold superior at 20 min as compared to S37 or *B. cinerea* separately. Thus, we might assume that S37 primes the oxidative burst in grapevine in the presence of *B. cinerea*.

#### Activation of MAP Kinases

A rapid activation of MAP kinases has been shown in different plant systems to mediate elicitor-induced defense responses (Gomez-Gomez and Boller, 2002; Zhang et al., 1998). The treatment of grapevine cells with *S. anulatus* S37, *B. cinerea*, and S37 + *B. cinerea* activated rapidly and transiently both 45 and 49 kD MAP kinases (**Figure 1C**). This activation was detected as soon as 15 min (**Figure 1C**), maintained at a high level for 30 min and reached the basal level around 60 min (data not shown).

### Analyzing Cell Death and Caspase-Like Activity

**FIGURE 2:**
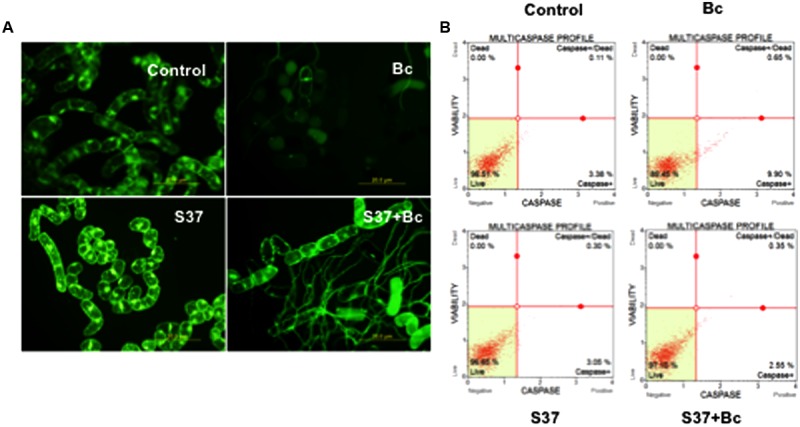
Monitoring cell death in grapevine cell suspensions by FDA as a probe **(A)** or by multicaspase profile **(B)** in control, cells challenged with *B. cinerea*, with S37, or with S37 + *B*. *cinerea.* Data presented are representative of triplicate experiments. Bc, *Botrytis cinerea;* S37, *S*. *anulatus* S37.

During the cell and microbes interaction, cells can be damaged, thereby affecting their viability. To further elucidate cell viability during the process of inoculation with *B. cinerea* and to study the impact of the presence of *S. anulatus* S37, grapevine cells were stained using fluorescein diacetate as a vital stain. Grapevine cells with intact plasma membranes and respiration revealed a green fluorescence while those without FDA-fluorescence were considered dead. Control cells showed strong FDA signals (**Figure 2A**), with high viability levels. While the presence of *S. anulatus* S37 in contact with cells has no impact on the viability, the FDA signal was weaker in cells infected with *B. cinerea*, indicating disturbed cell membranes and an inhibited respiratory activity. However, when cells were inoculated with S37 then with *B. cinerea*, the FDA signal was weak than the control but significantly higher than in cells infected solely with *B. cinerea*.

The multi-caspase activity was measured as described in “Materials and Methods.” The data in **Figure 2B** showed that this activity was not impacted significantly when grapevine cells suspension was brought into contact with S37. However, in the presence of *B. cinerea*, the percentage of cells with caspase activity increased significantly compared to the previous two terms (9.90%). When grapevine cells were previously inoculated with S37 before their infection with *B. cinerea*, this activity decreased significantly (2.55%).

### Expression of Defense-Related Genes

**FIGURE 3:**
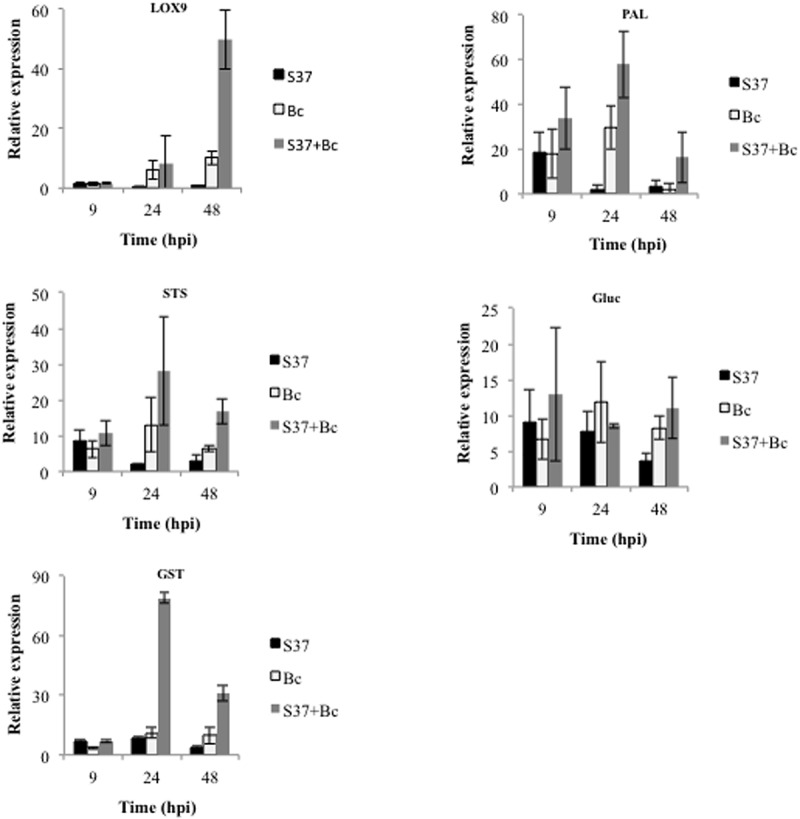
Relative expression of defense related genes in grapevine cell suspensions. Gene expression was monitored in control, cells challenged with *B*. *cinerea*, with S37, or with S37 + *B*. *cinerea.* Data presented are means of triplicate experiments ± SD. Bc, *Botrytis cinerea;* S37, *S*. *anulatus* S37; LOX9, lipoxygenase; PAL, phenylalanine ammonia-lyase; STS, stilbene synthase; Glue, glucanase; GST, Glutathione-*S*-transferase.

Expression profiles of number defense-related genes were analyzed by qRT-PCR in grapevine cells suspension after their inoculation with *S. anulatus* S37 and/or *B. cinerea*. Genes used correspond to those reported to be up-regulated during grapevine-microbe/MAMP interactions (Aziz et al., 2003; Varnier et al., 2009; Bordiec et al., 2011) and include those encoding a lipoxygenase (LOX9), a phenylalanine ammonia-lyase (PAL), a stilbene synthase (STS), a basic glucanase (Gluc), and a glutathione *S*-transferase (GST). All defense genes were up-regulated when the cells were inoculated with S37 or *B. cinerea* starting after 9 h except for *LOX9* (**Figure 3**).

The level of gene expression was lower in S37-treated cells compared to those infected with *B. cinerea*. Interestingly, inoculation with S37 + *B. cinerea* results in the priming of *LOX9* gene expression, which was almost 20 times higher than control. A priming effect was also seen for *PAL*, *STS*, and *GST*. The basic glucanase (*Gluc*) was the only gene, which was induced but not primed by S37 (**Figure 3**).

### Phytoalexin Production

**FIGURE 4:**
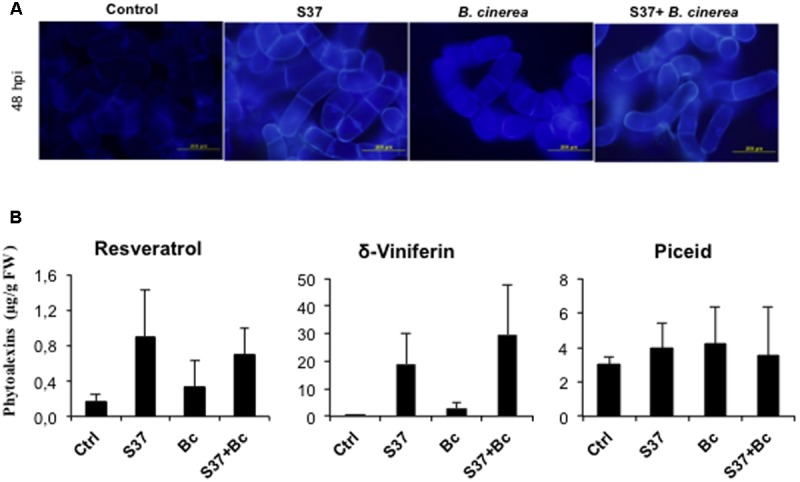
Production of phytoalexins by grapevine cells. **(A)** Autofluorescence microscopy under UV excitation of inoculated cells. Data presented are representative of triplicate experiments. **(B)** Phytoalexins, resveratrol δ-viniferin and piceid were measured at 48 h from control, cells challenged with S37, with *B*. *cinerea*, or with S37 + *B*. *cinerea.* Data are means from three replicates ± SD. Bc, *Botrytis cinerea*, S37, *S. anulatus* S37.

The resveratrol (*trans*-3,4′,5-trihydroxystilbene) and its dehydrodimer viniferin have been reported as the major phytoalexins produced by grapevine in response to the microbial infection and was associated with plant resistance to fungal pathogens (Adrian et al., 1997; Aziz et al., 2016). As shown in **Figure 4**, induction of resveratrol and *trans-*δ-viniferin was observed following treatment of grapevine cells with S37. A slight induction of both stilbenes was also observed after challenge with *B. cinerea*. Amounts of δ-viniferin accumulated were generally greater than resveratrol. However, with both microbes, the level of the glycosylated resveratrol, piceid, remained comparable to that of the control. With S37 + *B. cinerea*, the production of resveratrol and piceid showed similar levels than with S37, while δ-viniferin accumulation was primed.

### Refractory State

**FIGURE 5:**
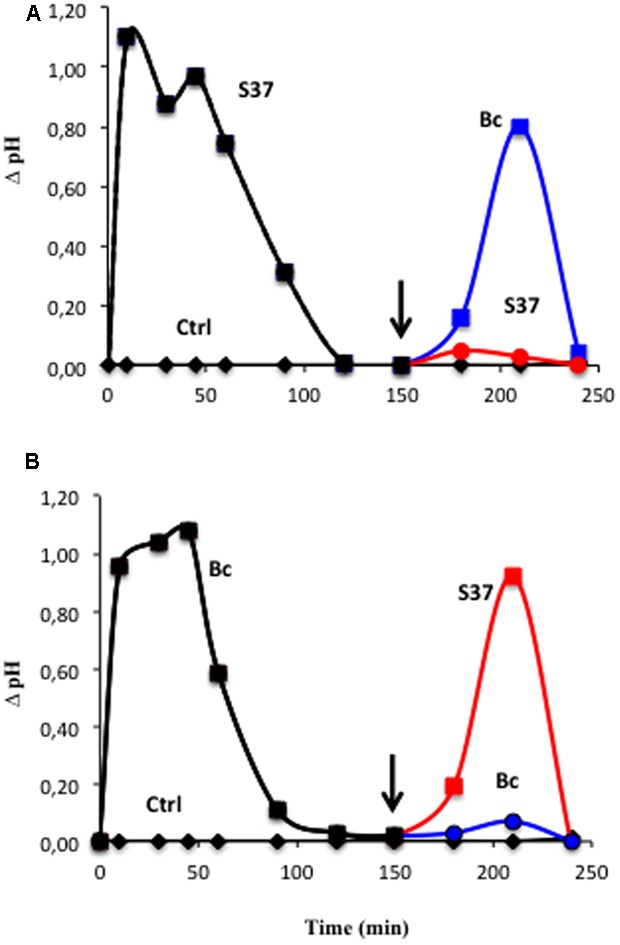
Refractory state experiments on grapevine cell suspensions monitored by extracellular pH measurement after successive inoculation with S37 and/or *B. cinerea.* Cells were first treated at time 0 with S37 **(A)** or Bc **(B)**, washed three times with fresh medium, then treated a second time (arrow) with S37 (read line) or with Bc (blue line). Data are means ± SD of duplicates, representative of two independent experiments. Bc: *Botrytis cinerea*; S37, *S. anulatus* S37.

One of the characteristics of cells is the occurrence of the refractory state, which often occurs after the perception of MAMPs/PAMPs at high-affinity protein receptors in the plasma membrane. As *S. anulatus* S37 and *B. cinerea* induce the same pattern of early events, refractory assays (i.e., the inability of the cells to react to a second application of the same elicitor), using extracellular pH were performed by successive additions of S37 and/or *B. cinerea*. Grape cells pretreated with the S37 were shown to be refractory to the second application of S37. However, they were not refractory to an application of *B. cinerea* (**Figure 5A**). Similarly, cells pretreated with *B. cinerea* were shown to be refractory to the second application of *B. cinerea*, while they were not refractory to an application of S37 (**Figure 5B**).

## Discussion

The *Streptomyces anulatus* S37 has been demonstrated as a protector of the grapevine against *B. cinerea* (Loqman et al., 2009), but molecular mechanisms involved in this interaction remains unknown. In this study, we attempt to decipher early events involved during the bacterial recognition and subsequent defense reactions after the *B. cinerea* challenge.

### *S. anulatus* S37 Induces Early Events in Grapevine Cells

Our results showed that *S. anulatus* S37 triggers the oxidative burst, one of the key events, in grapevine cell signaling. ROS play a crucial role either as toxic substances to halt the growth of the pathogen or as second messengers in the defense mechanism, leading to efficient plant protection (Torres, 2010; Zipfel and Robatzek, 2010). Nevertheless, only a few examples have described the involvement of ROS as a signal during the grapevine plants-beneficial bacteria interaction. For instance, *P. fluorescens* PTA-CT2, *P. aeruginosa* 7NSK2, and *B. subtilis* PTA-271 trigger a strong oxidative burst in grapevine cell suspensions (Verhagen et al., 2010, 2011). Authors suggested that the H_2_O_2_ production in grape cell suspensions could contribute as a signaling molecule to induce a disease resistance by these strains *in planta*. Indeed, although the accumulation of H_2_O_2_ is often associated with a characteristic plant early response following perception of pathogen avirulence signals (Lamb and Dixon, 1997), it was also shown that, in the symbiotic interaction with rhizobia, bacteria are initially recognized as intruders but then prevent or overcome plant defense responses (Santos et al., 2001). However, in contrast with our results, no significant variation in H_2_O_2_ levels was induced in grapevine cells by *B. phytofirmans* PsJN (Bordiec et al., 2011). Further, Van Loon et al. (2008) have reported that the production of H_2_O_2_ induced by *P. fluorescens* WCS417 was not matched with the expression of ISR in the whole tobacco against *Erwinia carotovora*.

The *S. anulatus* S37 also induces a strong extracellular alkalinization, which suggests an activation of plasma membrane H^+^-ATPases, to restore the pH gradient between the apoplast and the cytosol. This typical signature has been previously demonstrated during the microbial or MAMP perception (Aziz et al., 2004; Bordiec et al., 2011; Farace et al., 2015). The perception of S37 by grapevine cells was also associated with the phosphorylation of two mitogen-activated protein (MAP) kinases with relative molecular masses of 49 and 45 kDa. These effects resembled those achieved by the MAMPs in tobacco (Zhang et al., 1998) and in grapevine (Aziz et al., 2004; Trouvelot et al., 2008). MAP kinase cascades are major pathways downstream of sensors/receptors that transduce extracellular stimuli into intracellular responses in plants (Zhang et al., 1998). Their activation is known as an early physiological response to microbial recognition and plays a pivotal role in the plant innate immune system (Asai et al., 2002; Aziz et al., 2004; Meng and Zhang, 2013). In tobacco, two MAP kinases, designated SA-induced protein kinase (SIPK) and wounding-induced protein kinase (WIPK) are activated in a disease resistance-specific manner following pathogen infection or elicitor treatment (Kishi-Kaboshi et al., 2010). Similarly, it has been shown that lack of MPK3 increases the basal susceptibility of *Arabidopsis* to *B. cinerea*, while the lack of MPK6 suppresses flg22-induced resistance to *B. cinerea* (Galletti et al., 2011). It is noteworthy that these early responses induced by the *S. anulatus* S37 were not accompanied by a programmed cell death of grapevine, as shown by the FDA signal and the multicaspase activity. This result is in line with previous reports who indicated that oxidative burst or the activation of MAP kinases induced by beneficial bacteria are independent of hypersensitive cell death (Bargabus et al., 2003; Van Loon et al., 2008; Verhagen et al., 2010).

The Ca^2+^ influx is also involved in the microbial signal perception and can regulate many early responses in plant cells. In this context, we investigated whether extracellular alkalinization, H_2_O_2_ production, and MAPK activity depend on the calcium influx in grapevine cell suspensions. Using the EGTA, a Ca^2+^ influx inhibitor, the extracellular medium alkalinization was not impacted for all treatment, suggesting that this event does not depend on Ca^2+^-mediated pathways (Supplementary Figure S1). However, the high oxidative burst and MAPK activity induced by *S. anulatus* S37 and/or *B. cinerea* were completely abolished by EGTA in grapevine cells, suggesting that both microorganisms trigger H_2_O_2_ production and MAPK activity through Ca^2+^ dependent pathways. Similar results have been reported by Vandelle et al. (2006), using elicitors derived from *B. cinerea*. The possible regulation of the oxidative burst and the MAPK activation by the Ca^2+^ influx could result from a stimulation of NADPH-oxidase, the major enzymatic source of ROS, together with activation of Ca^2+^-dependent MAPK.

### *S. anulatus* S37 Induces the Defense-Related Genes Expression and Phytoalexin Accumulation

The treatment with S37 induced the upregulation of genes that are responsive to *B. cinerea* (Bézier et al., 2002), MAMPs (Aziz et al., 2004), and beneficial bacteria (Gruau et al., 2015). These include genes encoding secondary metabolism (PAL, STS, LOX9, GST), and the glucanase (PR2). However, we observed that the expression of all selected genes was lower in S37-treated cells compared to those infected with *B. cinerea*. This suggests that S37, as for other beneficial bacteria, can initially be perceived as a potential invader, resulting in the activation of the plant immune system (Zamioudis and Pieterse, 2012). These results are consistent with the hypothesis that plants respond weakly to beneficial microbes or derivative MAMPs to avoid a strong activation of defense responses that could be detrimental to fitness (Van der Ent et al., 2009). However, even the level of gene expression was low during the S37-grapevine cell interaction, it emphasizes the involvement of JA and SA signaling pathways upon the S37 perception. This was supported by an enhanced expression of *LOX9*, as a key element of the oxylipin synthesis, and an induced expression of *PR-2* (Gluc) and GST genes, which were found to be induced by SA in grapevine leaves (Dufour et al., 2013; Gauthier et al., 2014). The expression of these genes as JA- or SA-dependent responses was also responsive to *Pseudomonas fluorescens* PTA-CT2 and *Burkholderia phytofirmans* PsJN and as beneficial bacteria in grapevine tissues (Gruau et al., 2015; Miotto-Vilanova et al., 2016).

### *S. anulatus* S37 Primes Cell Responses after *B. cinerea* Inoculation

Our results show that grapevine cells infected with the *B. cinerea* exhibited similar early responses to those induced by the beneficial bacterium *S. anulatus* S37, except that the pathogen triggered the programmed cell death. This suggests the existence of some similarities and differences in signaling pathways involved in the recognition of beneficial and pathogenic microbes. However, the triggered cell death by *B. cinerea* might be a capital mechanism in the infection process with this necrotrophic pathogen (Gruau et al., 2015). As S37 and *B. cinerea* induced the same pattern for early events, we further performed desensitization assays using extracellular pH. We demonstrated that once increased by *S. anulatus* S37, the grapevine cells became refractory to further stimulation by *B. cinerea*, and inversely. This suggests a different mode of perception of the beneficial and pathogenic microbes by grapevine cells. Interestingly, upon a challenge with *B. cinerea*, *S. anulatus* S37 primed grapevine cells for enhanced defense reactions with a decline in the cell death. A higher production of H_2_O_2_ and an enhanced extracellular alkalinization were observed in S37-treated grapevine cells once challenged with *B. cinerea*. Since in this study S37 or *B. cinerea* do not produce H_2_O_2_ (data not shown), we might suggest that S37 primes the oxidative burst in grapevine cells as evident by the enhanced accumulation of H_2_O_2_ after pathogen challenge. *B. cinerea* itself has been shown to produce H_2_O_2_ in germinating conidia during the early steps of tissue infection (Heller and Tudzynski, 2011; Viefhues et al., 2014), or in response to CaCl_2_ exposure (Marschall and Tudzynski, 2016). However, the priming state of grapevine cells to produce more H_2_O_2_ without direct contribution of S37 or *B. cinerea* is consistent with our previous study showing that the bacterium S37 exerts an antifungal effect on *B. cinerea* by destructing its mycelium (Couillerot et al., 2014). In meanwhile, it cannot be excluded that the magnitude of the burst of H_2_O_2_ could be ascribed to different PAMP/MAMP compounds released by both the bacterium (Van Loon et al., 2008) and *B. cinerea* (Vandelle et al., 2006) in the medium. Furthermore, the time course of H_2_O_2_ burst is more in agreement with the timeline observed in grapevine cell cultures, but not in *B. cinerea*, after treatment with different MAMPs (Aziz et al., 2007), in which this response was linked to the expression of defense-related genes and development of leaf resistance against *B. cinerea*.

Alternatively, the low contribution of *B. cinerea*, if any, to the enhanced oxidative burst, could be due the low virulence of the strain used in this study. Indeed, it has been shown that ROS production by *B. cinerea* is an important component of its virulence, and increased levels of ROS in plant cells may contribute to host cell death and favors fungal infection (Heller and Tudzynski, 2011; Marschall and Tudzynski, 2016).

The level of activation of the 45 and 49 kDa MAP kinases was also maintained at high level in S37-treated cells after *B. cinerea* challenge. We may suggest, the MAP kinase activity is already at it maximum and no more activation was observed after subsequent exposure to pathogen. In accordance with our results, it has been reported that, in *Arabidopsis*, MP3 kinase was not primed, but rather linked to direct responses to pathogen infection and MAMP signaling (Nakagami et al., 2005). This is in line with the fact that both oxidative burst and activation of MAPK were considered as possible regulators of defense responses (Aziz et al., 2004; Beckers et al., 2009).

Several reports have suggested that most beneficial bacteria primed plants for the activation of various cellular defense responses upon the pathogen attack (Conrath et al., 2006; Van der Ent et al., 2009; Gruau et al., 2015). In this study, we further showed that the relative expression of some defense-related genes was upregulated by S37 after *B. cinerea* challenge. The expression of *LOX9* gene was almost 20 times higher in primed cells than in *B. cinerea*-infected ones. A priming effect was also seen with the expression of *PAL*, *STS*, and *GST* genes, while the basic glucanase (PR2) was not primed by the S37. Interestingly, the primed *PAL* and *STS* expressions were correlated with the S37-enhanced phytoalexin accumulation after *B. cinerea* challenge. These results are consistent with other previous researches showing that beneficial bacteria prime grapevine cells and leaves for an accelerated and an enhanced capacity to activate defense responses (Verhagen et al., 2011; Gruau et al., 2015), such as the rapid accumulation of hydrogen peroxide and phytoalexins as well as the activation of some defense-related genes upon the *B. cinerea* infection. Engineering the STS into plants of interest resulted in resveratrol accumulation and elevated pathogen resistance (Jeandet et al., 2013). A slight induction of stilbenic phytoalexins, resveratrol and its metabolic products, the glycoside piceid and the oxidized dimer δ-viniferin was observed after perception of S37, while only amount of δ-viniferin accumulated was greater after *B. cinerea* challenge. Similar results were observed in grapevine plants treated with *P. fluorescens* PTA-CT2 and *Pantoea agglomerans* PTA-AF2, which showed enhanced resistance to *B. cinerea* (Aziz et al., 2016). The accumulation of δ-viniferin in S37-treated cells indicated that this oligomer could be a possible marker for induced resistance to gray mold.

Overall, this study demonstrated for the first time that *S. anulatus* S37 induced a rapid and transient generation of H_2_O_2_, extracellular alkalinization and an activation of two MAPKs followed by a differential expression of some defense-related genes and a phytoalexin accumulation to lesser amounts, but not the programmed cell death. Interestingly, most of these defense responses were primed by the S37 after the pathogen challenge, with a decline in the cell death. Desensitization assays using the extracellular pH showed that once increased by the *S. anulatus* S37, cells became refractory to further stimulation by the *B. cinerea*, suggesting that grapevine cells distinctly perceived beneficial and pathogenic microbes.

## Author Contributions

PV-P and EAB designed the research. PV-P, EAB, AA, MR, SV, and HM carried out the experiments and analysis/interpretation of data. PV-P, AA, MR, and EAB wrote the manuscript with contributions and discussion from all of the co-authors. All authors have given approval to the final version of the manuscript.

## Conflict of Interest Statement

The authors declare that the research was conducted in the absence of any commercial or financial relationships that could be construed as a potential conflict of interest.
