# Supplementary material for: How Streptomyces anulatus Primes Grapevine Defenses to Cope with Gray Mold: A Study of the Early Responses of Cell Suspensions
Source: Front Plant Sci. 2017 Jun 28;8:1043. doi: 10.3389/fpls.2017.01043 (PMC5487444; doi:10.3389/fpls.2017.01043)
Supplement: Supplementary file 1 [file Table_1.PDF]

Supplementary Table S1: Primer sequences used for RT-PCR analysis of defense-related genes

Gene	Accession number	Forward Primer (5'-3')	Reverse primer (5'-3')	Annealing temperature (°C)	Amplicon size (bp)
PAL	XM_003635637.1	TCCTCCCGGAAAACAGCTG	TCCTCCAAATGCCTCAAATCA	59	101
STS	NM_001281117.1	AGGAAGCAGCATTGAAGGCTC	TGCACCAGGCATTTCTACACC	60	101
LOX9	NM_001281249.1; XM_002280615.1	CCCTTCTTGGCATCTCCCTTA	TGTTGTGTCCAGGGTCCATTC	60	101
GST	NM_001281248.1	TGCATGGAGGAGGAGTTCGT	CAAGGCTATATCCCCATTTTCTTC	60	98
Gluc	NM_001280967.1	TCAATGGCTGCAATGGTGC	CGGTCGATGTTGCGAGATTTA	59	155
EF1α	XM_002284888.1	AACCAAAATATCCGGAGTAAAAGA	GAACTGGGTGCTTGATAGGC	56	150
60RSP	XM_002270599.1	ATCTACCTCAAGCTCCTAGTC	CAATCTTGTCCTCCTTTCCT	55	166
