# Supplementary figures and images for: How Streptomyces anulatus Primes Grapevine Defenses to Cope with Gray Mold: A Study of the Early Responses of Cell Suspensions
Source: Front Plant Sci. 2017 Jun 28;8:1043. doi: 10.3389/fpls.2017.01043 (PMC5487444; doi:10.3389/fpls.2017.01043)

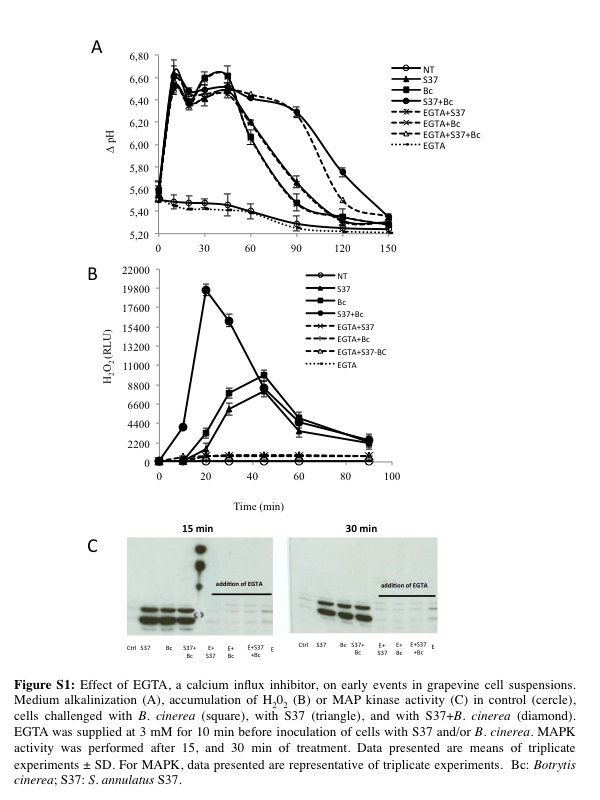

Supplement: Supplementary file 2 [file Image_1.TIFF]
